# Cooperativity of the SUMO and Ubiquitin Pathways in Genome Stability

**DOI:** 10.3390/biom6010014

**Published:** 2016-02-25

**Authors:** Minghua Nie, Michael N. Boddy

**Affiliations:** Department of Cell and Molecular Biology, The Scripps Research Institute, La Jolla, CA 92037, USA; nboddy@scripps.edu

**Keywords:** Ubiquitin, SUMO, STUbL, DNA damage

## Abstract

Covalent attachment of ubiquitin (Ub) or SUMO to DNA repair proteins plays critical roles in maintaining genome stability. These structurally related polypeptides can be viewed as distinct road signs, with each being read by specific protein interaction motifs. Therefore, via their interactions with selective readers in the proteome, ubiquitin and SUMO can elicit distinct cellular responses, such as directing DNA lesions into different repair pathways. On the other hand, through the action of the SUMO-targeted ubiquitin ligase (STUbL) family proteins, ubiquitin and SUMO can cooperate in the form of a hybrid signal. These mixed SUMO-ubiquitin chains recruit “effector” proteins such as the AAA^+^ ATPase Cdc48/p97-Ufd1-Npl4 complex that contain both ubiquitin and SUMO interaction motifs. This review will summarize recent key findings on collaborative and distinct roles that ubiquitin and SUMO play in orchestrating DNA damage responses.

## 1. Introduction

The integrity of the cell’s genome is under constant threat from various external and internal genotoxic agents, such as UV and oxidative stress. Unrepaired damage can lead to cell death or gene mutations that alter cellular behavior, potentially leading to the demise of the whole organism [1]. DNA damage can range from subtle changes in nucleotide base structure to breaks in both strands of the DNA double helix, and can occur in all phases of the cell cycle. Cells diligently scan their genome with damage sensor proteins and bring in specialized repair enzymes once a lesion is detected. Small lesions, such as simple nucleotide alterations on one strand of DNA can be repaired by base excision repair (BER) or nucleotide excision repair (NER). Extensive and difficult-to-repair DNA damage, such as double-strand breaks (DSBs) or replication forks stalled at DNA lesions, elicits a more extensive response called the DNA damage response (DDR), leading to increased production of repair enzymes and cell-cycle arrest until repair is complete [2].

ATM and ATR are key kinases that drive the DDR by phosphorylating chromatin, DNA repair and cell cycle checkpoint proteins at sites of DNA damage. In addition to phosphorylation, a plethora of other post-translational modifications (PTMs) are critical to the DDR. PTMs alter the chromatin environment as well as the interactions or activity of some repair enzymes to promote signal amplification, sequential recruitment, and removal of factors to allow for downstream repair steps. Using PTMs to modulate protein interactions and activity is a common and effective strategy to induce rapid and reversible responses. Proteins can be a modified by a small chemical moiety, such as the phospho- or acetyl-groups; or by another protein, such as members of the ubiquitin family [2,3,4].

Two structurally related small protein modifiers, ubiquitin and SUMO, are conjugated to target proteins via a similar cascade of biochemical reactions. Both SUMO and ubiquitin play important, often interconnected, and even interdependent roles in DNA damage repair. There are a number of excellent reviews discussing the roles of ubiquitin and SUMO in DNA repair published in the past few years [4,5,6,7,8,9,10,11]. This review will summarize some of the most recent advances in understanding the roles of ubiquitin and SUMO in DNA damage repair, with emphasis on the collaborative roles of these modifications.

## 2. Ubiquitin and SUMO Pathways, and Their Crosstalk

Covalent attachment of the carboxyl terminus of ubiquitin or SUMO (ubiquitylation or sumoylation) to a lysine residue on a substrate protein involves a similar relay of E1 activating, E2 conjugating, and E3 ligase actions [3]. Ubiquitylation and sumoylation are readily reversible, and deconjugation is carried out by ubiquitin- or SUMO-specific iso-peptidases, such as DUBs or Ulp/SENPs. Thus, just like phosphorylation, SUMO and ubiquitin are highly dynamic modifiers of protein behavior.

**Figure 1:**
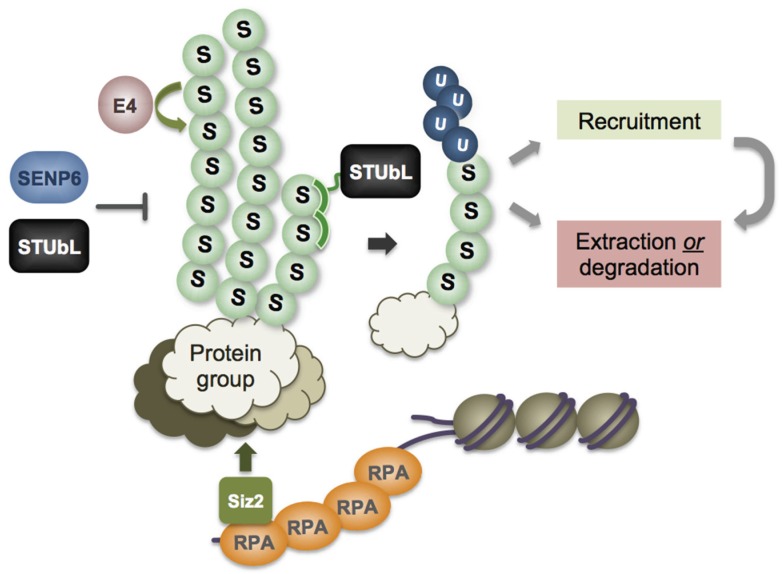
DNA damage promotes recruitment of sumoylation enzymes and crosstalk between the SUMO and ubiquitin pathways. In the process of repairing a DSB, resection generates ssDNA, which is coated with RPA. Through interaction with RPA, SUMO E3 ligase Siz2 is recruited to the damage site to promote the sumoylation of protein groups involved in DNA repair. SUMO-SIM contacts create a glue effect to enhance protein-protein interactions and the recruitment of additional factors.

Crosstalk between the SUMO and ubiquitin pathways had been documented, and in general was of an antagonistic nature [12]. This made the discovery of the STUbL (SUMO-Targeted Ubiquitin Ligase) family of proteins all the more intriguing. STUbLs, including mammalian RNF4, budding yeast Slx5-Slx8, and fission yeast Rfp1/2-Slx8 heterodimers, can direct SUMO conjugates for degradation at the proteasome [13,14,15,16,17]. In this mechanism, a tandem array of SUMO interaction motifs (SIMs) in STUbLs enables them to selectively ubiquitylate target proteins that are modified by polymeric SUMO chains (Figure 1). Thus, in addition to their distinct signaling roles, SUMO and ubiquitin cooperate in the processing of certain target proteins. Additional examples of crosstalk between SUMO and ubiquitin pathways will be given throughout this review.

SUMO chains can form, assisted by multiple SIM-containing E4 elongases. SUMO chains may then attract the tandem SIM-containing ubiquitin E3 ligase, STUbL, which adds ubiquitin to the SUMO chain to produce ubiquitin-SUMO hybrid chains, a signal that is “read” by proteins containing both a ubiquitin interacting motif (UIM) and a SIM. These hybrid chains can either recruit additional repair factors, or promote protein extraction from complexes/chromatin through the activity of the AAA^+^ ATPase complex Cdc48/p97-Ufd1-Npl4. STUbL can also curb SUMO chain elongation by capping the SUMO chain with ubiquitin. SUMO chains can also be actively antagonized by SUMO isopeptidases, such as SENP6. Genotoxins such as CPT promote the dissociation of SENP6 from chromatin, therefore shifting the balance toward more sumoylation at the damage site. Crosstalk between the SUMO and ubiquitin pathways has been demonstrated on Top1, ID complex, Rad52, and XPC extraction.

## 3. DNA Damage-Induced Recruitment of SUMO and Ubiquitin E3 Ligases

Ubiquitin pathway enzymes far out-number those found in the SUMO pathway, even though both are involved in diverse cellular activities. It is thought that the SUMO pathway achieves substrate specificity in part by regulating the subcellular localization of sumoylation/desumoylation enzymes. For example, the PIAS family and Nse2/Mms21 SUMO E3 ligases can associate with the genome by interacting with DNA and/or chromatin-bound proteins. The PIAS family proteins, including mammalian PIAS1-4, budding yeast Siz1/2, and fission yeast Pli1, contain a SAP (Scaffold attachment factor, Acinus and PIAS) domain, which directly binds DNA. The DNA repair process can further promote interaction between the SUMO E3 ligase and damaged chromatin (Figure 1). For example, RPA-coated ssDNA generated by resection is required for Siz2 accumulation at DNA DSBs and local Siz2-mediated sumoylation of DNA repair factors [18]. Mechanistically, the SIZ2 SAP domain extension (eSAP) interacts directly with the middle subunit of the RPA complex, Rfa2. Interestingly, neither DNA binding by the core SAP domain, nor SUMO interaction via the SIM of SIZ2, is required for its recruitment to DSBs.

The Nse2/Mms21 SUMO E3 ligase does not contain a SAP domain, but is part of the chromatin-associated Smc5-Smc6 complex [19,20,21,22]. How Smc5-Smc6, and thereby Nse2, is recruited to chromatin and/or DNA lesions is generally poorly defined. Fission yeast Smc5-Smc6 accumulates during S phase at the heterochromatic centromeres, and redistributes to subtelomeres in an Nse2 SUMO E3 ligase-dependent manner following DNA alkylation (MMS) damage [23]. This indicates a role for Smc5-Smc6 mediated sumoylation in its own accrual at DNA damage sites. Moreover, the Smc5-Smc6 subunit Nse1 contains an E3 ubiquitin ligase RING domain that also supports Smc5-Smc6 accumulation at MMS-induced DNA lesions [24,25]. It should be noted that an initial analysis of Nse1 indicated that by itself it did not support *in vitro* E3 ubiquitin ligase activity [24]. However, a more recent study revealed that Nse1 must be in complex with a MAGE domain cofactor (MAGE-G1/Nse3) to exhibit E3 ubiquitin ligase activity in standard *in vitro* assays [26].

**Figure 2:**
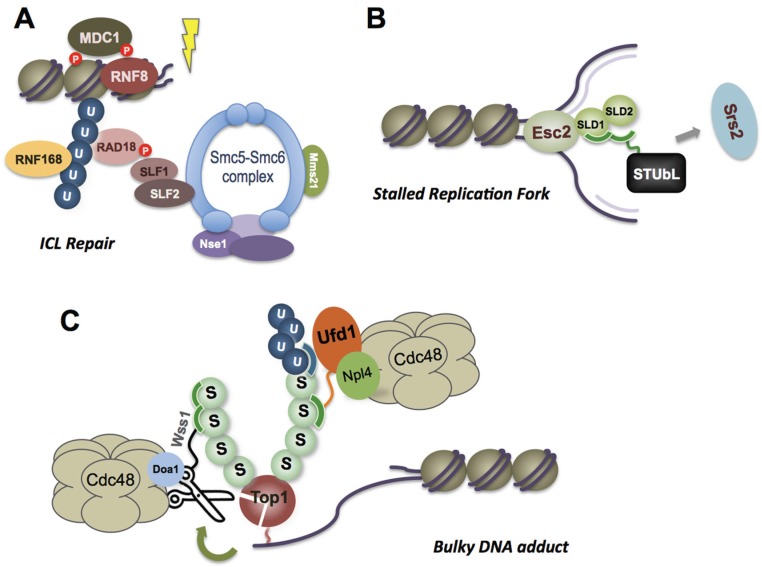
Examples of the Integration of SUMO and Ubiquitin Signaling. (**A**) Ubiquitin and SUMO E3s Nse1 and Nse2 are part of the chromatin-associated Smc5-Smc6 complex. Certain lesions such as ICLs induce the recruitment of Smc5-Smc6 to DNA damage sites via the interaction of ubiquitin chains, generated by RNF168, with RAD18 and the Smc5-Smc6 subunits SLF1 and SLF2. “P” indicates phosphorylation of DDR factors, including RAD18, that is also required for Smc5-Smc6 recruitment; (**B**) DNA at stalled replication forks may directly bind the SUMO mimic Esc2, which via its integral SUMO-like domains (SLDs) is suggested to attract STUbL to facilitate the proteolytic removal of anti-recombinase Srs2; (**C**) SUMO chains are added to a Top1-DNA adduct and/or nearby factors to signal STUbL-mediated ubiquitylation and subsequent extraction/degradation facilitated by the Cdc48/p97-Ufd1-Npl4 complex. In addition, in budding yeast the SUMO chain can recruit a SIM-containing metalloprotease Wss1, which is activated by ssDNA. Wss1 also cooperates with a Cdc48 complex, containing another cofactor Doa1.

Therefore, cooperativity between sumoylation and ubiquitylation exists within the same complex, potentially acting as a switch for relocalization of Smc5-Smc6 in response to genotoxic insults (such cooperativity will be further discussed below). Despite these correlative results, the targets and mechanisms of Smc5-Smc6-mediated sumoylation or ubiquitylation in its localization to DNA lesions remain undefined. Notably, human Smc5-Smc6 was recently shown to localize at certain DNA lesions via an interaction network that is based on RNF168-catalyzed ubiquitin chains [27]. These ubiquitin chains are bound by the E3 ubiquitin ligase RAD18, which is proposed to act structurally and not catalytically in this role due to the absence of its cofactor Rad6 (Figure 2A). Finally, Rad18 recruits Smc5-Smc6 via a phospho-dependent interaction with the SLF1 subunit of the SLF1/SLF2 loading factor ([27]; Figure 2A). A role for human Nse2 or Nse1 E3 ligase activities in loading remains to be tested.

Once at DNA damage sites SUMO E3 ligases locally sumoylate DDR proteins, which promotes interactions amongst them via SUMO-SIM contacts and creates a “protein glue” effect. For example, the checkpoint protein ATRIP is recruited to RPA-coated ssDNA where it becomes SUMO-2/3 modified. ATRIP sumoylation promotes interaction of multiple ATRIP-associated proteins, including ATR, RPA70, TopBP1, and the MRN complex, which all have affinity for SUMO2 chains [28].

The division of labor among SUMO E3 ligases in the DDR differs between organisms. Fission and budding yeast PIAS mutants (*pli1∆* & *siz1∆/siz2∆*) are not overtly sensitive to genotoxins, whereas Nse2/MMS21 SUMO E3 ligase-deficient cells are hypersensitive to a broad spectrum of DNA damaging agents [29,30]. Human PIAS and Nse2 SUMO E3 ligases both contribute to the DDR, albeit through largely independent targets [21,31,32,33]. Similarly, *Drosophila* dPIAS and Nse2 independently influence the repair of DSBs in heterochromatin [34].

In addition to local SUMO E3 ligase recruitment, regulated SUMO deconjugation enhances the sumoylation of certain DDR targets. The SUMO deconjugating enzyme SENP6 dissociates from RPA70 in the presence of camptothecin (CPT) causing RPA^SUMO−2/3^ to accumulate, which facilitates Rad51 recruitment to CPT-induced damage sites [35].

## 4. Potential New SUMO E3 Ligases in DNA Repair

Besides their catalytic SP-RING domains, PIAS SUMO E3 ligases contain a single SIM that may help fuel target sumoylation *in vivo* by recruiting conjugation competent SUMO~Ubc9. Recently, *RING-independent* SUMO E3 ligases that utilize multiple SIMs to catalyze sumoylation have been described [36,37,38,39,40,41]. DNA repair proteins SLX4 and Wss1 both have multiple SIMs [38,39,42]. SLX4 is a large multi-domain protein that scaffolds and localizes key DNA endonucleases to support a number of DNA repair pathways. As with other multi-SIM containing proteins, SLX4 SIMs interact with SUMO chains. Distinguishingly however, SLX4 SIMs also mediate selective interaction with the charged E2 enzyme Ubc9~SUMO, thereby promoting autosumoylation and the sumoylation of the SLX4-interacting endonuclease subunit, XPF [39]. SLX4’s SIMs are also required for its localization to certain DNA structures/lesions [39,42,43], where it may cooperate with PIAS-type ligases such as PIAS3 in sumoylating key DDR factors [32].

Wss1 is important for UV damage repair [44], and was recently found to proteolytically remove bulky DNA adducts such as topoisomerase I stalled in its cleavage complex by CPT [38,45] (Figure 2C). Interestingly, as determined for STUbL in fission yeast (see #6 below, [46]), Wss1 also cooperates with the Cdc48/p97 molecular segregase in Top1 lesion repair [38,45]. Wss1 protease activity is activated *in vitro* by ssDNA, and *in vivo* by genotoxic stress, in part due to its oligomerization. Like SLX4, Wss1 may exert SUMO E3 ligase activity via its tandem SIM and promote SUMO chain formation [38]. While SLX4 has been shown to interact preferentially with Ubc9~SUMO [39], such evidence is currently lacking for Wss1. Both factors may also/instead facilitate SUMO chain formation by providing a structural scaffold to nucleate and activate the sumoylation machinery; an E4 elongase activity. Binding of Wss1 to these self-catalyzed SUMO chains might, like ssDNA [38], promote its oligomerization and local activation.

The vertebrate ZNF451 protein was recently defined both structurally and biochemically as a novel factor with both E3 ligase and E4 elongase activities [40,41]. Importantly, the tandem SIMs of ZNF451 elongate SUMO2/3 chains catalytically (E4), in contrast to the SUMO chain-binding SIMs present in the STUbL RNF4 [41]. This indicated the presence of unique features in ZNF451 SIMs. Indeed, the ZNF451 inter-SIM linker domain contains a PLRP motif that is catalytically critical, and together with the first SIM binds Ubc9~SUMO2 [40,41]. The SUMO2 E3 ligase activity of ZNF451 also requires a zinc finger, which likely recruits the first SUMO2 (substrate) for subsequent SIM-dependent elongation. These analyses of ZNF451 underscore the need for caution previously noted about SIM-containing factors masquerading as SUMO ligases *in vitro* [47]. Whether ZNF451 is a key player in the DDR is currently unknown; however, genotoxin-induced SUMO2/3 conjugation is globally reduced in ZNF451 deleted cells [41]. Given the apparently pivotal role played by SUMO2/3 chains in the DDR, it appears likely that ZNF451 activity will impact DNA repair.

## 5. SUMO Mimicry Integrates SUMO and Ubiquitin Signaling in DNA Repair

Rad60 of fission yeast is the founding member of a conserved protein family with two integral SUMO-like domains (SLDs) that plays key roles in genome stability [48,49]. These Rad60 family roles include mimicking SUMO to interact with SIM-containing factors such as STUbLs, and the SUMO conjugating enzyme Ubc9 [50,51,52,53]. The latter interaction promotes target sumoylation by the Smc5-Smc6-associated SUMO ligase Nse2/Mms21 in the highly evolutionarily diverged fission and budding yeasts [51,54].

Notably, fission yeast STUbL, Nse2 and Rad60 cooperate to repair stalled Topoisomerase I cleavage complexes (Top1cc) in cells lacking the Top1cc-resolving enzyme Tdp1 [55]. This was the first example of STUbL, Smc5-Smc6 and Rad60 acting in concert to promote DNA repair, and recent analyses are supporting broad conservation of this phenomenon. In budding yeast, the Slx5-Slx8 STUbL was shown to antagonize Nse2/Mms21-dependent SUMO conjugates, indicating they also have overlapping targets [54,56]. In addition, the budding yeast Rad60 family protein Esc2 binds DNA at stalled replication forks to regulate the proteolysis of the helicase and anti-recombinase Srs2, which it appears to achieve by recruiting Slx5-Slx8 to ubiquitylate Srs2 (Figure 2B) [57]. Moreover, Smc5-Smc6 and Esc2 share overlapping roles in regulating replication stress-associated recombination [58,59].

Esc2-dependent Slx5-Slx8 localization to replication forks may be an example of SUMO-independent STUbL recruitment. There is a precedent for such STUbL recruitment, in the recognition and ubiquitylation of unmodified budding yeast Matα2 by Slx5-Slx8 [60,61]. However, Rad60 is modified by SUMO [62], and so a similar modification of Esc2 could enhance STUbL recruitment at replication forks.

A recent striking example of cooperativity between STUbL, Smc5-Smc6 and Rad60 is the relocalization of DNA breaks within *Drosophila* heterochromatin to the nuclear periphery to undergo repair, which requires all three factors [34]. Compromising this relocalization pathway renders cells sensitive to genotoxic stress, such as ionizing radiation [34]. As these studies were done with knockdowns of each factor, which of the spectrum of functions of dRad60, STUbL and Smc5-Smc6 involved awaits a more detailed analysis of hypomorphic alleles.

Of note, relocalization of at risk genomic loci/lesions to the nuclear periphery in budding yeast also depends on the Slx5-Slx8 STUbL [63,64]. Based on the cooperativity of Smc5-Smc6, STUbL and Rad60 family proteins in DNA repair in fission yeast and *Drosophila*, it will be interesting to determine the role for budding yeast Esc2 and Smc5-Smc6 in this phenomenon. In this regard, Smc5-Smc6 was shown to promote relocalization of a DNA DSB in the rDNA locus of budding yeast to an extranucleolar site for repair [65]. Thus, although currently piecemeal, aggregate data in budding yeast supports the conservation and key nature of a cooperative Smc5-Smc6, STUbL and Rad60 (Esc2) DNA repair axis.

The only other example of SUMO mimicry is found in vertebrate UAF1, which also contains tandem SLDs [66]. The USP1/UAF1 complex deubiquitylates the Fanconi anemia protein FANCD2, which promotes homologous recombination (HR) and DNA cross-link repair. UAF1 SLD2 binds directly to a SIM in FANCI, bridging the interaction between USP1/UAF1 and FANCD2/FANCI. It is not known if UAF1 SLDs make additional contacts with the SUMO machinery, or are restricted to SIM:SUMO-like interactions.

## 6. Recruitment Mechanisms and Functions of STUbL on Chromatin

There are a number of excellent recent reviews covering many of the E3 ubiquitin ligases that are recruited to chromatin/DNA (e.g., [4,10]), so here we focus largely on the recruitment mechanisms and functions of the STUbL family. Examples of ubiquitin E3 ligases that directly contact DNA/chromatin are RNF168, RNF4 and BMI1-RING1B, which bind DNA and/or the nucleosome, and the UV-DDB complex that recognizes UV damaged DNA [10,67,68]. Several RING finger domain-containing ubiquitin E3s also belong to the SF2-helicase superfamily that translocate on DNA in an ATP-driven manner. For example, the activity of yeast Rad5 (human HLTF and SHPRH) and STUbL Uls1 (also known as Ris1) lead to ubiquitylation (and removal in the case of Uls1) of chromatin-associated proteins, such as PCNA and Rap1, and as such act like a “molecular sweeper” [10,69]. Notably, most of these E3 ligases also contain interaction motifs for SUMO, ubiquitin or other PTMs, supporting combinatorial and thus more selective recognition of their targets (see below and [4]).

The mammalian STUbL RNF4 binds to various DNA structures as well as nucleosomes [67,68]. RNF4 targets the nucleosome via a basic cluster in its RING domain, similar to RNF168 and RING1b-BMI1 [67]. Nucleosome targeting by RNF4 is important for repairing TRF2-depleted telomeres via 53BP1 recruitment and NHEJ [67]. Moreover, as for its recruitment to DNA DSBs, RNF4’s SIMs are critical for telomere repair, suggesting that interactions of RNF4 with both SUMO and nucleosomes cooperatively promote DNA repair [67,70,71,72].

STUbLs appear to have multiple functions at DNA damage sites (Figure 1). As SUMO E3s are brought to the damage site to catalyze local sumoylation, heightened local accumulation of SUMO could lead to uncontrolled growth of SUMO chains. While SUMO chains may promote interaction of repair proteins at the damage site, unrestricted growth could also hinder the downstream repair process. By attaching ubiquitin to the SUMO amino terminus [73], STUbL can both stop the growth of SUMO chains and target them for proteasome-mediated protein degradation. Notably, fission yeast STUbL mutant’s genotoxin sensitivity is fully suppressed in cells that are unable to form SUMO chains, indicating that a major genome protective function of STUbL is to prevent SUMO chain accumulation [46,51].

STUbL activities also generate hybrid ubiquitin- and SUMO-chains, which function as a unique signal to attract proteins that contain recognition motifs for both modifications [46,74]. Interaction between a hybrid Ub-SUMO chain and its corresponding recognition motifs can lead to further protein recruitment. For example, RAP80 contains both a UIM and SIM that bind hybrid chains to stabilize RAP80’s residence at DSBs and facilitate the recruitment of BRCA1 [74]. On the other hand, Ub-SUMO chains can also promote protein extraction from complexes/chromatin and/or degradation, as in the case of Cdc48/p97-Ufd1-Npl4 segregase recruitment (Figure 1) [4,46,75,76,77]. For both RAP80 and Ufd1, integral ubiquitin- and SUMO-recognition motifs synergize in protein interaction as they bind hybrid ubiquitin/SUMO chains more avidly than homogenous chains of either modifier ([74] and personal observation).

## 7. Coordinated and Distinct Roles of Ubiquitin and SUMO in DNA Repair

As discussed above, SUMO and ubiquitin modifications can cooperatively facilitate both DNA repair factor recruitment and removal at lesions (see also [4]). In both scenarios, the presence of both modifications improves binding affinity, specificity, or both. Below are examples of recently characterized coordinated and distinct roles of ubiquitin and SUMO in DNA repair.

Two central components of the Fanconi anemia (FA) repair pathway, FANCI and FANCD2 (the ID complex), are sumoylated in response to DNA damage [78]. FANCI sumoylation has also been observed in several proteomics studies [71,79,80]. ID complex sumoylation is mediated by PIAS1/4, and antagonized by SENP6. ID complex sumoylation at DNA damage sites again requires the concerted action of PTMs, in this case phosphorylation by ATR and monoubiquitylation, which together license the chromatin loading of ID. Subsequently, ID sumoylation promotes its polyubiquitylation by RNF4 and extraction from chromatin by the p97/Cdc48 segregase complex. In contrast, a sumoylation defective ID complex is refractory to RNF4, p97, and SENP6 regulation. Thus, the cooperative role of STUbL and Cdc48/p97 initially defined in fission yeast [46], also has critical functions in human genome stability [78]. Overall, multiple PTMs and members of the SUMO pathway (PIAS1/4, SENP6, and STUbL) work in concert to fine-tune the balance of activated ID complex at DNA lesions.

Using SUMO to fine-tune the balance between repair factor recruitment and removal likely represents a common strategy used in multiple DNA repair pathways. HR is a key process used in DNA DSB repair, interstrand crosslink (ICL) removal, and Post-Replicative Repair (PRR). During HR, invasion of homologous double-stranded DNA (dsDNA) by a 3’-single stranded DNA (ssDNA) requires the coating of ssDNA with recombinase Rad51 to form a nucleoprotein filament. The loading of Rad51 is promoted by Rad52, which displaces RPA from ssDNA while assisting Rad51 binding.

In budding yeast, Rad52 is sumoylated upon DNA damage by Siz2 [81]. Rad52 sumoylation is enhanced by ssDNA [82], likely due to recruitment of Siz2 by RPA coated ssDNA generated during DSB resection [18]. A Rad52 mutant refractory to sumoylation appears impaired in its interaction with SIM-containing cofactor Rad51 [83]; although Rad52 sumoylation has only subtle effects on HR *in vivo* [81,82,84]. This is a recurrent theme in SUMO biology, where multiple components within a pathway are decorated by SUMO, possibly providing signaling redundancy.

In addition to, or instead of promoting HR, Rad52 sumoylation can curb Rad51 loading by attracting the Cdc48/p97-Ufd1-Npl4 molecular segregase complex through a SIM in Ufd1 [75,83]. The Cdc48 complex then drives the dissociation of Rad52-SUMO and Rad51 from DNA [83]. Notably, genotoxic stress such as HU or MMS can also induce the Slx8-dependent degradation of Rad52-SUMO, suggesting that Rad52-SUMO dissociated by Cdc48 might also be subject to STUbL-mediated ubiquitylation [64].

UV induced DNA damage is detected by the sensor proteins DDB2 and XPC. Following UV radiation, DDB2 and XPC are ubiquitylated by the Cul4A/B E3 ligase [85]. Whereas ubiquitylated DDB2 is rapidly degraded via p97/Cdc48-Ufd1-Npl4 and UBXD7-mediated mechanisms, XPC ubiquitylation enhances its interaction with DNA [86]. XPC is also sumoylated, and XPC sumoylation promotes both global genome NER (ggNER) and UV-induced ubiquitylation of XPC [87]. Although RNF4 is recruited to laser-induced DSBs through SUMO-SIM interactions and is important for DSB repair, it is dispensable for UV-induced ubiquitylation of XPC. Therefore, SUMO-SIM interaction is not the sole factor in STUbL target selection. Instead, another SIM-containing STUbL called RNF111/Arkadia mediates the UV-induced ubiquitylation of sumoylated XPC [88]. XPC ubiquitylation (K63 chain topology) by RNF111 promotes XPC’s release from UV-lesions, and thus more efficient recruitment of downstream NER factors such as XPG and XPF/ERCC1 [89].

In contrast to the SUMO and ubiquitin recognition motifs of RAP80 and Ufd1 that synergize to interact with Ub-SUMO hybrid chains, those in SLX4 appear to direct it into distinct DNA repair pathways. SLX4 is a platform that interacts with multiple classes of repair proteins, including SLX1, XPF^Rad1^/ERCC1^Rad10^, the MUS81-EME1 Holliday Junction (HJ) resolvase, the MSH2-MSH3 mismatch repair complex, the telomeric protein TRF2, and the mitotic control kinase PLK1 [90]. As anticipated from this scaffolding role, SLX4 is key for multiple DNA repair pathways, including ICL repair and proper chromosome segregation in the absence of the RecQ helicase *BLM* [91,92,93,94]. SLX4’s ubiquitin and SUMO interacting motifs direct SLX4 to ICL and replicative stress at Common Fragile Sites (CFS), respectively [39,42]. Therefore, the coincidence of SUMO and ubiquitin interaction motifs does not necessarily indicate affinity for Ub-SUMO hybrid chains. It is likely that the particular juxtaposition of these motifs in a protein, together with other PTMs and protein interaction domains, dictates their functionality.

## 8. Conclusions and Future Directions

SUMO and ubiquitin modifications critically orchestrate the DDR in both time and nuclear space. At DNA damage sites, both modifiers promote the rapid recruitment of DDR proteins. Just as crucial for DNA repair and genome stability, recent discoveries have revealed direct cooperation between SUMO and ubiquitin in chromatin remodeling at DNA lesions. Certain SUMO targets are ubiquitylated by STUbLs, thereby attracting Cdc48/p97-Ufd1-Npl4 or other effectors with affinity for Ub-SUMO hybrid chains. This chromatin remodeling machinery, coupled to recycling or proteasomal degradation of extracted targets, is likely to be a broadly used and key mechanism in the dynamic control of chromatin constituents.

Key questions remaining include, how is selectivity generated in the STUbL pathway? That is, RNF4 and RNF111 STUbLs both preferentially bind SUMO chains, but nevertheless have distinct SUMO conjugated targets. Perhaps subnuclear compartmentalization of STUbLs plays a role, as it does in generating target specificity of the desumoylating enzymes ULP1/2 (human SENP1/2). Regulation of STUbLs by additional PTMs such as phosphorylation is also likely, to license their activities on particular targets.

To derive general “rules” for STUbL and other E3 ligase targeting to DNA lesions, chromatin mass spectrometry (CHROMASS; [27]) in combination with comprehensive PTM analysis to identify repair factors and their modifications at various stages of a DNA repair process would be informative.
